# TAK-242, a specific inhibitor of Toll-like receptor 4 signalling, prevents endotoxemia-induced skeletal muscle wasting in mice

**DOI:** 10.1038/s41598-020-57714-3

**Published:** 2020-01-20

**Authors:** Yuko Ono, Yuko Maejima, Masafumi Saito, Kazuho Sakamoto, Shoichiro Horita, Kenju Shimomura, Shigeaki Inoue, Joji Kotani

**Affiliations:** 1 Department of Disaster and Emergency Medicine, Graduate School of Medicine, Kobe University, Kobe, 650-0017 Japan; 2 Department of Bioregulation and Pharmacological Medicine, School of Medicine, Fukushima Medical University, Fukushima, 960-1295 Japan; 3 Department of Bio-Informational Pharmacology, School of Pharmaceutical Sciences, University of Shizuoka, Shizuoka, 422-8526 Japan

**Keywords:** Biochemistry, Biochemistry, Biochemistry, Proteolysis, Proteolysis

## Abstract

Circulating lipopolysaccharide (LPS) concentrations are often elevated in patients with sepsis or various endogenous diseases related to bacterial translocation from the gut. Systemic inflammatory responses induced by endotoxemia induce severe involuntary loss of skeletal muscle, termed muscle wasting, which adversely affects the survival and functional outcomes of these patients. Currently, no drugs are available for the treatment of endotoxemia-induced skeletal muscle wasting. Here, we tested the effects of TAK-242, a Toll-like receptor 4 (TLR4)-specific signalling inhibitor, on myotube atrophy *in vitro* and muscle wasting *in vivo* induced by endotoxin. LPS treatment of murine C2C12 myotubes induced an inflammatory response (increased nuclear factor-κB activity and interleukin-6 and tumour necrosis factor-α expression) and activated the ubiquitin–proteasome and autophagy proteolytic pathways (increased atrogin-1/MAFbx, MuRF1, and LC-II expression), resulting in myotube atrophy. In mice, LPS injection increased the same inflammatory and proteolytic pathways in skeletal muscle and induced atrophy, resulting in reduced grip strength. Notably, pretreatment of cells or mice with TAK-242 reduced or reversed all the detrimental effects of LPS *in vitro* and *in vivo*. Collectively, our results indicate that pharmacological inhibition of TLR4 signalling may be a novel therapeutic intervention for endotoxemia-induced muscle wasting.

## Introduction

Sepsis, defined as a life-threatening condition caused by a dysregulated response to infection^1^, is a leading cause of death and disability in intensive care units. Recent estimates suggest that the global incidence of sepsis is 31.5 million cases per year, resulting in about 5.3 million deaths annually^2^. Moreover, the incidence of sepsis is gradually increasing, placing a tremendous economic burden on society^2–4^. Lipopolysaccharide (LPS), a component of the outer cell membrane of Gram-negative bacteria, is a principal mediator in the pathophysiology of Gram-negative bacterial sepsis^5^. Plasma levels of LPS are often elevated in patients with sepsis or septic shock caused by Gram-negative bacteria, Gram-positive bacteria, or fungus^5^, related to bacterial translocation from the intestinal tract to the circulation^6^. Increased levels of circulatory endotoxin and persistent systemic inflammation are also observed in various endogenous diseases, including diabetes mellitus^7^, cancer^8^, liver cirrhosis^9^, chronic heart failure^10^ and end-stage kidney disease^11^, a phenomenon known as metabolic endotoxemia.^12,13^ Severe involuntary loss of skeletal muscle, termed muscle wasting, is present in all these conditions, suggesting a potential role for endotoxemia and inflammation in its development^12,13^. Skeletal muscle wasting contributes to generalized weakness and debilitation, worsens survival and functional outcomes, and increases societal health care costs. For example, skeletal muscle weakness associated with sepsis is a powerful independent predictor of prolonged length of mechanical ventilation, intensive care unit and hospital stay, hospital and 1-year mortality^14–16^, and long-term physical disabilities^17^. Thus, there is an urgent need to advance therapeutic interventions for endotoxemia-induced skeletal muscle wasting.

Previous studies^18–20^ have shown that LPS-induced skeletal muscle wasting is primarily caused by proteolysis related to a systemic inflammatory response to infection. Two major cellular proteolytic systems are known to be involved in endotoxemia-associated muscle weakness; namely, the ubiquitin–proteasome degradation pathway^18–20^ and the autophagy recycling pathway^19^. Expression of the E3 ubiquitin ligases atrogin-1/MAFbx and MuRF1 are upregulated in the muscles of animals with sepsis induced by caecal ligation and puncture^18^ or LPS administration^19,20^. In agreement with these experimental findings, increased ubiquitin–proteasome activity has been found in muscle biopsies from patients with sepsis^21^. Similarly, LPS treatment upregulated autophagosome formation and promoted atrophy in differentiated mouse C2C12 myotubes and mouse tibialis anterior (TA) muscles^19^. We recently confirmed and extended these observations by showing that LPS decreased the myogenic capacity of C2C12 myoblasts and induced myotube atrophy *in vitro*^22^.

Despite its debilitating effects, there is currently no approved drug for the treatment of endotoxemia-associated muscle weakness. Toll-like receptor 4 (TLR4), the cellular ligand for LPS, plays a central role in innate immunity and the response to infection^23^. TLR4-mediated signalling results in activation of the transcription factor nuclear factor-κB (NF-κB) and production of proinflammatory cytokines such as tumour necrosis factor (TNF)-α and interleukin (IL)-6. These factors are well-known modulators of protein turnover in muscle and contribute to the development of muscle atrophy in various inflammatory conditions^24–26^. TLR4 is abundantly expressed on skeletal muscle cells in humans and mice^27,28^, and circulating LPS readily penetrates peripheral tissues, including the extremities^29^. LPS–TLR4 signalling therefore may directly promote skeletal muscle inflammation^22,30,31^.

TAK-242, a specific inhibitor of TLR4 signalling, inhibited MyD88 and TRIF-dependent pathways by binding to Cys747 in the intracellular domain of TLR4^32–35^. Previous studies showed that TAK-242 prevented acute kidney injury and lung injury in LPS-injected sheep and mice^36,37^. Importantly, TAK-242 has been tested in a clinical trial of patients with sepsis and was found to be generally well tolerated^38^. However, there have been no previous reports of the impact of pharmacological TLR4 inhibition on skeletal muscle wasting and function during endotoxemia. In this study, we postulated that the pharmacological inhibition of TLR4 by TAK-242 may alleviate LPS-induced muscle wasting by suppressing activation of inflammatory and proteolytic pathways, such as ubiquitin–proteasome degradation and autophagy, in muscle cells. Utilising *in vitro* and *in vivo* approaches, we found evidence that supports our hypothesis and suggests that TLR4 inhibition warrants further investigation as a therapeutic strategy for endotoxemia-associated muscle weakness.

## Results

### TAK-242 inhibits LPS-induced myofibrillar protein loss and atrophy in C2C12 myotubes

**Figure 1:**
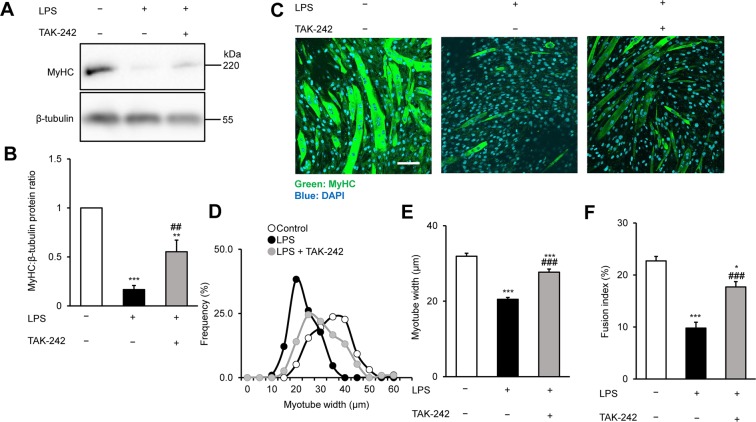
TAK-242 reduces LPS-induced atrophy of C2C12 myotubes. (**A,B**) Western blot analysis (**A**) and quantification (**B**) of MyHC expression in C2C12 myotubes treated for 48 h with vehicle, LPS (1 μg/mL), or LPS and TAK-242 (1 μM). Data in (**B**) were normalised to β-tubulin protein levels, and the ratio in vehicle-treated control cells was set to 1. N = 5/group. Full-length blots are presented in Supplementary Figure S4. (**C**) Representative immunofluorescence staining of MyHC in C2C12 myotubes treated as described for (**A,B**). Scale bar = 100 μm. (**D–F**) Distribution of myotube widths (**D**), mean myotube width (**E**), and fusion index (**F**) of C2C12 cells treated as described in (**A,B**). Myotube width was measured as described in the Methods. N = 97–114. The fusion index was calculated from five randomly selected fields as described in the Methods. For all panels, data are presented as the mean ± s.e.m. ***p < 0.001, **p < 0.01, *p < 0.05 vs vehicle control; ^###^p < 0.001, ^##^p < 0.01 vs LPS-treated group. P-values were derived from one-way ANOVA followed by Tukey’s honest significant difference test or Kruskal-Wallis test followed by Dunn’s post hoc tests with Bonferroni correction.

We previously found that TLR4 is constitutively expressed in mouse C2C12 myoblasts and myotubes^22^, indicating that LPS can directly induce myotube atrophy without involvement of the immune system, which is a major source of proinflammatory cytokines. To determine whether the pharmacological inhibition of TLR4 signalling can ameliorate LPS-induced muscle protein loss and atrophy in cultured myotubes, we analysed the expression of myofibre-specific myosin heavy chain (MyHC) and the size and proportion of mature C2C12 myotubes after culture for 48 h with vehicle, LPS (1 μg/mL), or LPS plus TAK-242 (1 μM). We found that MyHC protein expression was strongly downregulated by LPS, as previously noted by Doyle *et al*.^19^, but this was partially reversed by pretreatment with TAK-242 (Fig. 1A, B). Similarly, immunofluorescence staining of myotubes with an MyHC-specific antibody revealed that exposure to LPS reduced the diameter and abundance of mature myotubes, which was also rescued by TAK-242 pretreatment (Fig. 1C–F). These results indicated that inhibition of TLR4 signalling suppressed LPS-induced muscle protein loss and atrophy in myotubes.

### TAK-242 inhibits LPS-induced inflammatory and proteolytic pathways in C2C12 myotubes

**Figure 2:**
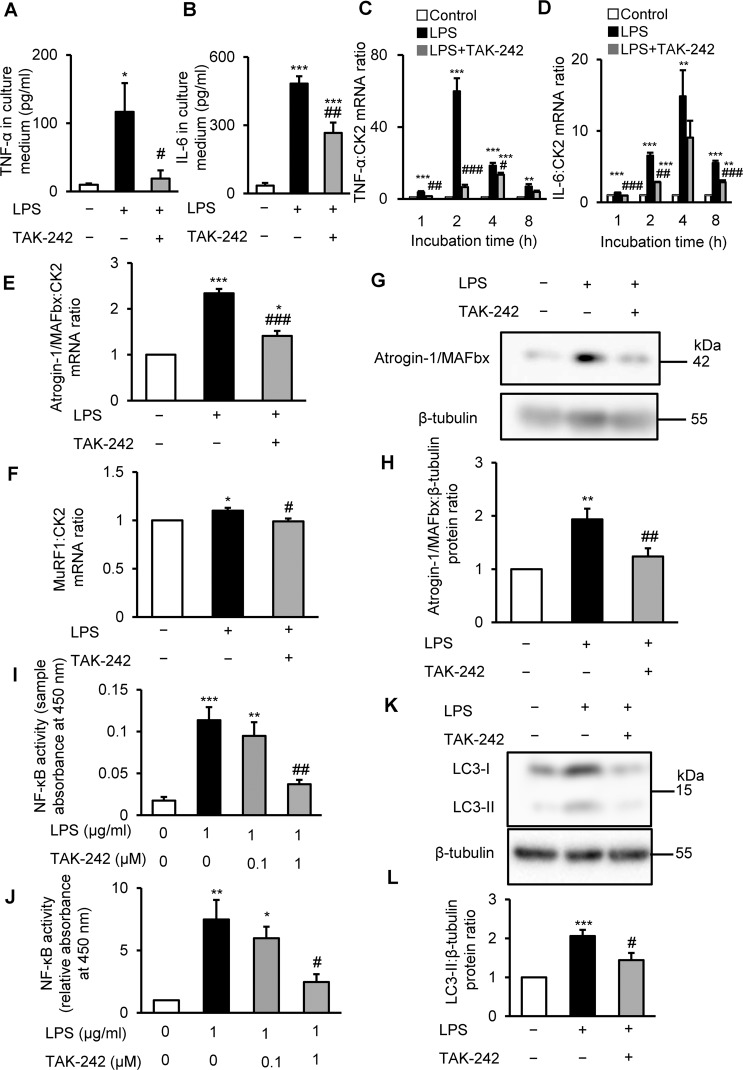
TAK-242 suppresses LPS-induced activation of inflammatory and proteolytic pathways in C2C12 myotubes. (**A,B**) C2C12 myotubes were treated with vehicle (0.1% vol/vol DMSO) or TAK-242 (1 μM) and then with PBS or LPS (1 μg/mL) 1 h later. After 4 h, cell culture supernatant was collected and TNF-α (**A**) and IL-6 (**B**) concentrations were measured by ELISA. N = 6/group. (**C,D**) qRT-PCR analysis of TNF-α (**C**) and IL-6 (**D**) mRNA levels in C2C12 myotubes treated for up to 8 h as described in (**A,B**). N = 4/group. (**E,F**) qRT-PCR analysis of atrogin1/MAFbx (**E**) and MuRF1 (**F**) mRNA in C2C12 myotubes treated for 3 h. N = 4–5/group. (**C–F**) Data were normalised to CK2 mRNA levels and are shown as fold increase over the vehicle-treated controls. (**G**,**H**) Western blot analysis (**G**) and quantification (**H**) of Atrogin-1/MAFbx in C2C12 myotubes treated for 4 h. Data were normalised to β-tubulin protein levels, and the ratio in vehicle-treated control cells was set at 1.0. N = 7/group. Full-length blots are presented in Supplementary Figure S5A. (**I,J**) NF-κB (p65) binding activity in C2C12 myotubes treated for 4 h with vehicle, LPS (1 μg/mL), or LPS plus TAK-242 (1 μM and 0.1 μM). NF-κB (p65) DNA-binding activity in myotubes was analysed using a TransAM ELISA kit. Data are shown as sample absorbance at 450 nm (**I**) or fold increase (**J**) over the vehicle-treated controls. N = 4/group. (**K,L**) Western blot analysis (**K**) and quantification (**L**) of LC3-II expression in C2C12 myotubes treated for 24 h. Data were normalised to β-tubulin protein levels, and the ratio in vehicle-treated control cells was set at 1.0. N = 14/group. Full-length blots are presented in Supplementary Figure S5B. All panels, data are presented as the mean ± s.e.m. ***p < 0.001, **p < 0.01, *p < 0.05 vs vehicle control; ^###^p < 0.001, ^##^p < 0.01, ^#^p < 0.05 vs LPS-treated group. P-values were derived from one-way ANOVA followed by Tukey’s honest significant difference test or Kruskal-Wallis test followed by Dunn’s post hoc tests with Bonferroni correction.

LPS is known to increase the expression of proinflammatory cytokines, such as TNF-α and IL-6, in mouse myotubes and skeletal muscle^27,30,31^, and these catabolic cytokines induced muscle proteolysis and atrophy^25,26,39^. Therefore, we investigated the effect of TLR4 inhibition on TNF-α and IL-6 expression in C2C12 myotubes treated for 4 h with vehicle, LPS (1 μg/mL), or LPS plus TAK-242 (1 μM). As expected, LPS elevated TNF-α and IL-6 levels in the culture medium, but pretreatment with TAK-242 significantly abolished the increased levels of these catabolic cytokines (Fig. 2A,B). Consistent with these findings, TAK-242 pretreatment significantly suppressed the increased mRNA levels of both cytokines in LPS-treated myotubes (Fig. 2C,D).

From these observations, we speculated that TAK-242 reduced the expression of inflammatory cytokines to restore the myofibrillar protein loss and atrophy in LPS-treated C2C12 myotubes. If this is true, the neutralisation of inflammatory cytokines should also ameliorate LPS-induced MyHC protein loss. To test this hypothesis, C2C12 myotubes were treated for 48 h with vehicle, LPS (1 μg/mL), or LPS and TNF-α neutralizing antibody (5 μg/mL). This concentration of antibody was used based on relevant previous findings^22,40^. As expected, the TNF-α neutralizing antibody partially but significantly reversed the LPS-induced downregulation of MyHC protein (Supplementary Fig. S1).

To further investigate the LPS-induced atrophy of myotubes, we measured two muscle-enriched ubiquitin ligases, atrogin-1/MAFbx and MuRF1, which are key enzymes in the ubiquitin–proteasome degradation pathway, a known contributor to endotoxemia or sepsis associated muscle atrophy^18–21^. Previous studies^18–21^ showed that atrogin-1/MAFbx and MuRF1 were upregulated in cultured muscle cells and mouse skeletal muscle in response to LPS. We found that TAK-242 pretreatment significantly reduced the mRNA expressions of both ubiquitin ligases in C2C12 myotubes treated with LPS for 3 h (Fig. 2E,F), which was previously found to be the optimal time point for the LPS-induced upregulation of atrogin-1/MAFbx and MuRF1 in these cells^18^. To extend these findings at the protein level, we measured atrogin-1/MAFbx protein expression in LPS and TAK-242 treated C2C12 myotubes. In agreement with the findings in Fig. 2E, western blot analysis showed that atrogin-1/MAFbx protein expression was upregulated in C2C12 cells after 4 h of LPS treatment, but this was partially rescued by pretreatment with TAK-242 (Fig. 2G,H).

NF-κB is a key transcription factor in inflammation and has been shown to mediate the increased expressions of cytokines and ubiquitin–proteasome pathway genes^24,25^. Therefore, we next examined the effects of LPS and TAK-242 on NF-κB (p65) activation in C2C12 myotubes using a specific ELISA. As shown in Figs. 2I and 2J, TAK-242 dose-dependently suppressed NF-κB DNA-binding activity in LPS-treated cells. These results collectively indicate that TLR4 signalling activates the NF-κB-induced expression of proinflammatory and proteolytic genes and proteins in cultured myotubes.

In addition to the ubiquitin–proteasome pathway, autophagy plays a crucial role in cellular proteolysis and has been implicated in various conditions associated with muscle atrophy^41^, including sepsis^19^. To evaluate the effect of LPS and TAK-242 on autophagy, we examined the expression of LC3-II, an indicator of autophagosome formation^42^. Western blot analysis showed an increase in LC3-II levels in C2C12 cells after 24 h of LPS treatment, in accordance with previous observations^19^, and this was abolished to baseline levels by preincubation with TAK-242 (Fig. 2K,L). Collectively, these results demonstrate that TAK-242 inhibits the TLR4–LPS-induced stimulation of the two major proteolytic pathways in C2C12 myotubes.

### TAK-242 fails to rescue the reduced activities of growth signals in LPS-treated C2C12 myotubes

Impairment of cell growth signals is another important determinant of skeletal muscle depletion. Among the growth signals that control muscle size, the PI3K/Akt pathway plays a pivotal role in promoting protein synthesis and blocking degradation^39,43^. Akt activates S6 kinase (S6K) via mammalian target of rapamycin, which leads to an increase in protein synthesis^39,43^. Previous studies showed that LPS markedly decreased muscle protein synthesis and growth in cultured muscle cells and skeletal muscles of rodents via the impaired phosphorylation of Akt and S6K^44–46^. Therefore, next we examined the effect of TAK-242 on the PI3K/Akt pathway in LPS-treated C2C12 myotubes. In agreement with previous findings^44–46^, the phosphorylation of p70 S6 kinase (Thr389) and Akt (Ser473) were decreased in C2C12 cells after 4 h of LPS treatment (Supplementary Fig. S2). However, TAK-242 pretreatment did not restore the decreased phosphorylation of these proteins in LPS-treated myotubes (Supplementary Fig. S2). Taken together, the preventive effect of TAK-242 on LPS-induced myotube atrophy was more likely related to the amelioration of proteolytic pathways rather than protein synthesis and cell growth pathways.

### TAK-242 prevents muscle wasting and weakness in LPS-treated mice

**Figure 3:**
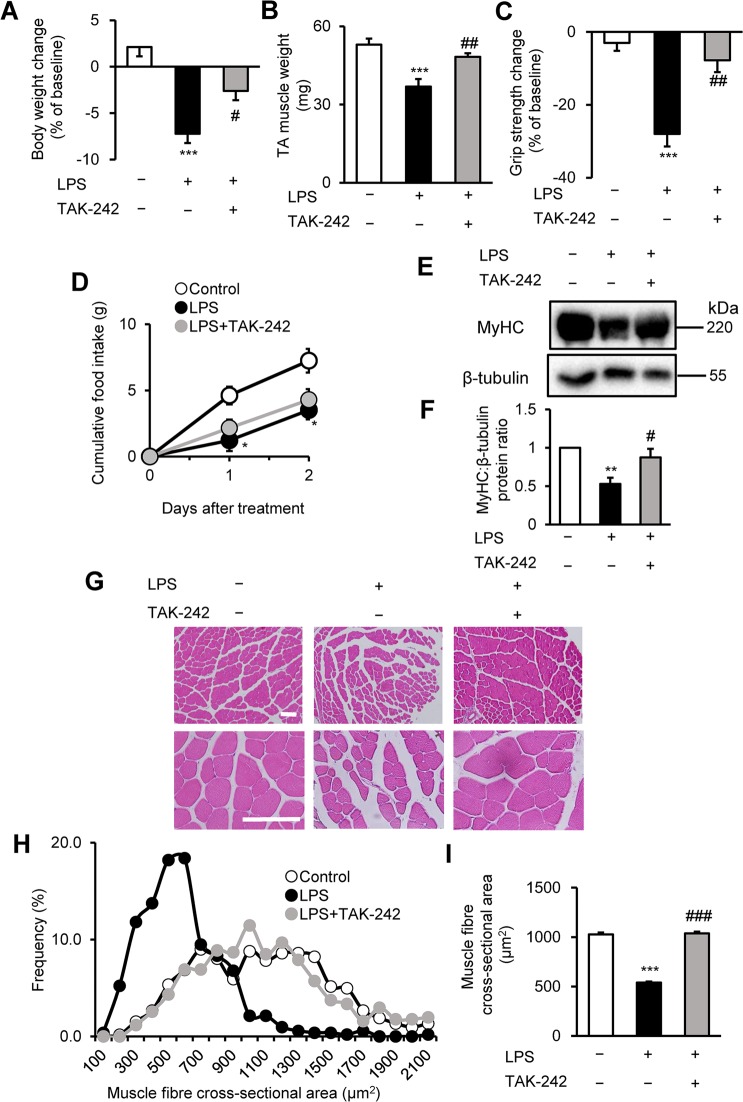
TAK-242 reduces LPS-induced muscle atrophy and weakness in mice. (**A**–**D**) Wild-type C57BL/6 mice (8–12-week-old males) were injected with vehicle (PBS containing 0.9% DMSO) or TAK-242 (3 mg/kg) and then with PBS or LPS (1 mg/kg) 1 h later. After 2 days, mice were assessed for body weight (**A**), TA muscle weight (**B**), and grip strength (**C**). Food intake (**D**) was measured every 24 h up to 48 h. N = 4–5/group. **(E**,**F**) Western blot analysis (**E**) and quantification (**F**) of MyHC expression in TA muscles at 2 days after administration of LPS (1 mg/kg) and TAK-242 (3 mg/kg) as described for (**A–D**). Data were normalised to β-tubulin protein levels, and the ratio in vehicle control-treated mice was set at 1.0. N = 7–8/group. Full-length blots are presented in Supplementary Figure S6. (**G–I**) Representative images of H&E-stained TA muscle sections (**G**) and quantification of the distribution (**H**) and mean (**I**) cross-sectional areas of TA muscle fibres at 2 days after administration of LPS (1 mg/kg) and TAK-242 (3 mg/kg) as described for (**A–D**). The cross-sectional area of TA muscle fibres was measured as described in the Methods. Scale bar, 100 μM. N = 506–523/group. For all panels, data are presented as the mean ± s.e.m. ***p < 0.001, **p < 0.01, *p < 0.05 vs vehicle control, ^###^p < 0.001, ^##^p < 0.01, ^#^p < 0.05 vs LPS-treated group by one-way ANOVA followed by Tukey’s honest significant difference test.

To extend these observations *in vivo*, we used a mouse model of endotoxemia-associated muscle weakness. For these experiments, male C57BL/6 mice (8–12 weeks of age) were treated with vehicle or TAK-242 (3 mg/kg) 1 h before the intraperitoneal (i.p.) injection of LPS (1 mg/kg) or phosphate-buffered saline (PBS). Two days later, we evaluated body weight, muscle mass, and muscle strength (grip strength assay). Notably, while LPS had a significantly detrimental effect on each of these parameters, TAK-242 pretreatment markedly and significantly reversed the LPS-induced body weight loss (Fig. 3A), TA muscle loss (Fig. 3B), and muscle strength loss (Fig. 3C) compared with vehicle-pretreated mice. At the end of the experiment, mice in the vehicle control group showed normal behaviour, with an adequate intake of food. In contrast, mice in the LPS injected group were lethargic and had lost their appetite for food (Fig. 3D). The effects on muscle mass and strength were further confirmed by western blot analysis, which revealed that TAK-242 prevented the LPS-induced loss of MyHC protein in TA muscles (Fig. 3E,F). In addition, histological examination of TA muscle sections showed that TAK-242 reversed the LPS-induced shrinkage of muscle fibres and increased the interstitial space (Fig. 3G–I) compared with vehicle-pretreated mice.

### TAK-242 blocks systemic catabolic cytokine release and skeletal muscle proteolysis in LPS-administered mice

**Figure 4:**
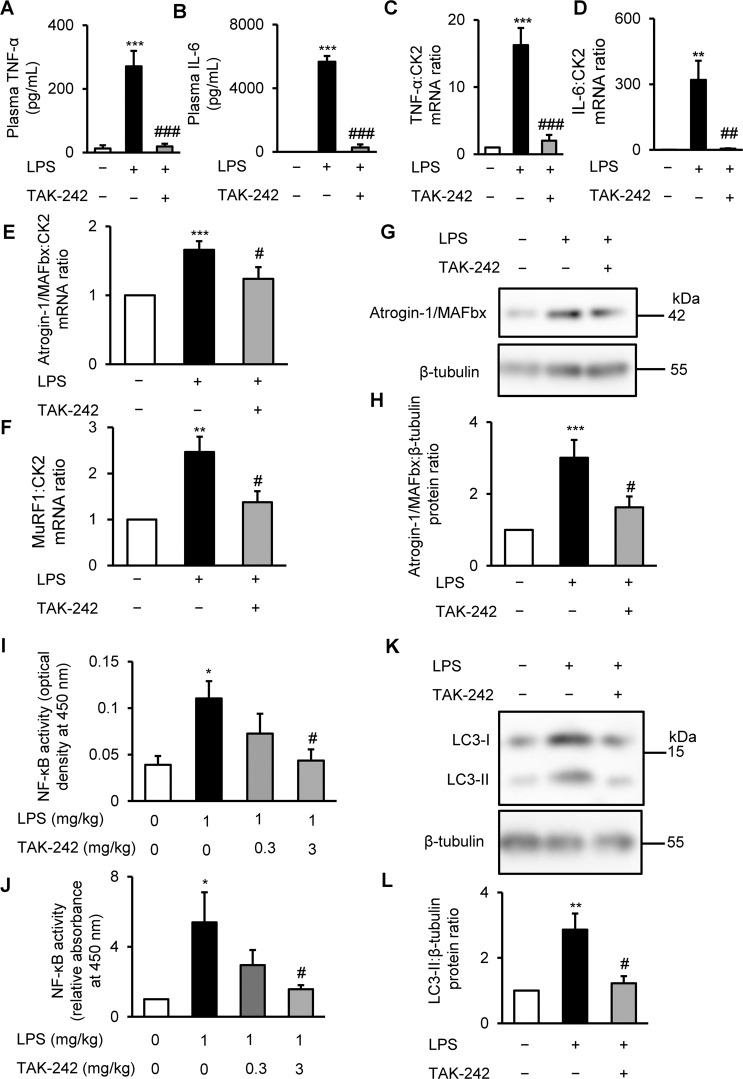
TAK-242 reduces LPS-induced inflammatory and muscle proteolytic pathways in mice (**A**,**B**) Wild-type C57BL/6 mice (8–12-week-old males) were injected with vehicle (PBS containing 0.9% DMSO) or TAK-242 (3 mg/kg) and then with PBS or LPS (1 mg/kg) 1 h later. After 4 h, plasma samples were prepared and TNF-α (**A**) and IL-6 (**B**) concentrations were measured by ELISA. N = 3–4/group. (**C–F**) qRT-PCR analysis of TNF-α (**C**), IL-6 (**D**), atrogin-1/MAFbx (**E**), and MuRF1 (**F**) mRNA in TA muscles at 4 h after administration of LPS (1 mg/kg) and TAK-242 (3 mg/kg). Data were normalised to CK2 mRNA levels and are shown as fold increase over the vehicle-treated controls. N = 5–6/group (**C**,**D**), or 4–9/group (**E**,**F**). (**G**,**H**) Western blot analysis (**G**) and quantification (**H**) of atrogin-1/MAFbx in TA muscles at 4 h after administration of LPS (1 mg/kg) and TAK-242 (3 mg/kg). Data were normalised to β-tubulin protein levels, and the ratio in vehicle control-treated mice was set at 1.0. N = 8/group. Full-length blots are presented in Supplementary Figure S7A. (**I,J**) NF-κB (p65) DNA-binding activity in TA muscles at 4 h after administration of LPS (1 mg/kg) and TAK-242 (3 mg/kg and 0.3 mg/kg) was analysed using a TransAM ELISA kit. Data are shown as sample absorbance at 450 nm (**I**) or fold increase (**J**) over the vehicle-treated controls. N = 7–9/group. (**K,L**) Western blot analysis (**K**) and quantification (**L**) of LC3-II expression in TA muscles at 24 h after administration of LPS (1 mg/kg) and TAK-242 (3 mg/kg). (**L**) Data were normalised to β-tubulin protein levels, and the ratio in vehicle control-treated mice was set at 1.0. N = 5–8/group. Full-length blots are presented in Supplementary Figure S7B. For all panels, data are presented as the mean ± s.e.m. ***p < 0.001, **p < 0.01, *p < 0.05 vs vehicle control; ^###^p < 0.001, ^##^p < 0.01, ^#^p < 0.05 vs LPS-treated mice by one-way ANOVA followed by Tukey’s honest significant difference test.

To determine whether the LPS-induced muscle weakness observed *in vivo* was mediated by the TLR4-stimulated activation of proinflammatory and proteolytic pathways, we analysed the cytokine levels and pathway activation in plasma and TA muscle samples from treated mice. Plasma TNF-α and IL-6 levels were markedly elevated in mice treated for 4 h with LPS, in agreement with previous observations^30,32^, but pretreatment with TAK-242 largely abolished this response (Fig. 4A,B). Consistent with the findings in plasma, TNF-α and IL-6 mRNA levels in TA muscles were significantly reduced in mice pretreated with TAK-242 compared with tissues from animals administered LPS alone (Fig. 4C,D).

Further analysis of the TA muscle extracts for effects on proteolytic pathways revealed that TAK-242 significantly reversed the effects of LPS on atrogin-1/MAFbx and MuRF1 mRNA levels (Fig. 4E,F), atrogin-1/MAFbx protein levels (Fig. 4G,H), NF-κB (p65) DNA-binding activity (Fig. 4I,J), and LC3-II levels (Fig. 4K,L). The effect of TAK-242 on the impaired growth signals in TA muscles of LPS-administered mice was also limited (Supplementary Fig. S3). These results were similar to the responses of cultured C2C12 myotubes treated with LPS and TAK-242. Collectively, these observations suggest that the pharmacological inhibition of TLR4 effectively prevents endotoxin-induced skeletal muscle wasting by reducing the activation of systemic and skeletal muscle inflammatory and proteolytic pathways, including ubiquitin–proteasomal degradation and autophagy.

## Discussion

The present study demonstrated that the pharmacological inhibition of TLR4 reduced skeletal muscle inflammatory and proteolytic pathway activation and reversed muscle wasting in LPS-treated cultured C2C12 myotubes and in mice with endotoxemia. Our finding that blockade of the TLR4–NF-κB signalling pathway reversed skeletal muscle loss suggests a potential new strategy for clinical intervention in skeletal muscle weakness induced by endotoxemia and subsequent systemic inflammation.

The results of our *in vitro* experiments demonstrate that LPS directly engages TLR4 on muscle cells to activate inflammatory and catabolic pathways, resulting in atrophy. Notably, these data indicate that LPS affects muscle cells independently of the immune system. Our findings are in line with earlier observations by Doyle *et al*.^19^ and Frost *et al*.^27^. However, our findings extend these studies by providing evidence that pharmacological inhibition of the TLR4–NF-κB axis inhibits skeletal muscle inflammation, reverses major proteolytic pathway activation, and reduces the atrophy of myotubes *in vitro* and skeletal muscle *in vivo*. In addition to sepsis, LPS is often detected in the circulation of elderly subjects^47^ and patients with pro-catabolic conditions such as chronic heart failure^10^, diabetes^7^, obesity^7^, cancer^8^, liver cirrhosis^9^, and end-stage kidney disease^11^. In these conditions, increased LPS levels are mainly caused by bacterial translocation from the intestinal tract to the circulation. Increased ubiquitin–proteasome activity and skeletal muscle wasting have been observed in all of these conditions^12,13^, and skeletal muscle expression of TLR4 is upregulated in many of them, including diabetes^28^, obesity^28^, end-stage kidney disease^48^, and advanced age^47,49^, indicating that the TLR4–NF-κB pathway may be elevated in these populations. Pharmacological inhibition of TLR4 by TAK-242 therefore might be especially beneficial at reversing skeletal muscle depletion in these populations. We also previously showed that TAK-242 ameliorated the LPS-induced impairment of C2C12 myogenesis^22^. Because reduced myogenic capacity^50^ and muscle protein catabolism^51,52^ are important determinants of skeletal muscle wasting, our previous and present data collectively suggest that LPS may directly affect skeletal muscle and induce muscle atrophy *via* synergistic effects on myogenesis and proteolysis.

TLR4 signalling involves the MyD88 and TRIF dependent pathways^23^. It was previously shown in macrophages that LPS-induced autophagy is regulated through a TRIF-dependent and MyD88-independent TLR4 signalling pathway^53^. Another study also showed that the muscle-specific deletion of MyD88 did not protect against muscle protein loss induced by LPS^54^. In a cancer cachexia model, TRIF was required for induction of the catabolic genes, *Mafbx, Murf1*, and *Foxo1* in skeletal muscle^55^. Doyle *et al*. also demonstrated that p38 MAPK was responsible for the LPS-induced activation of the ubiquitin-proteasome and the autophagy-lysosomal pathways in muscle^19^, which are downstream components of the TRIF dependent pathway. Taken together, we speculated that the catabolic effect of LPS in this study is likely to be mediated by the TRIF-dependent TLR4 signalling pathway.

Although elevated protein degradation accompanies muscle atrophy induced by fasting^52^, and denervation and disuse^24^, LPS-induced muscle wasting displays some distinctive features, including the presence of a systemic inflammatory response. Several clinical studies have identified distinct patterns of elevated circulating cytokines in patients with sepsis and septic shock^56–58^, and our finding of increased plasma TNF-α and IL-6 levels in the mouse model is consistent with these clinical data. Taken together, these observations suggest that systemic inflammatory reactions and the direct engagement of muscle-expressed TLR4 by pathogen-derived molecules may synergise to induce muscular wasting in sepsis. Interestingly, TLR4 inhibitors have shown beneficial effects on endotoxemia-associated dysfunction of other organs, including acute kidney injury in LPS-injected sheep^36^ and suppression of LPS-induced acute lung injury in mice^37^, suggesting that TLR4 inhibition may be an effective treatment for other complications of endotoxemia in addition to skeletal muscle wasting.

Currently, there are no approved drugs for the treatment of sepsis-associated muscle weakness. In addition to TAK-242, curcumin^59^, salicylate^24^, and meloxicam^60^ have been tested for their effects on sepsis-associated muscle weakness in animal models. These compounds were found to have anti-inflammatory activity and to inhibit NF-κB; however, high doses were required to achieve these effects, which may not translate well to clinical settings. In addition, curcumin is available only as an oral nutritional supplement. Of note, the dose of TAK-242 used in the animal study presented here was previously found to be well tolerated after its intravenous administration to patients with sepsis in an earlier clinical trial^38^.

Despite its beneficial effects on survival in animal models of sepsis^32,61,62^, clinical trials of TAK-242^38^ and other TLR4 antagonists^63^ have failed to observe a significant improvement of the 28-day mortality rate of patients with sepsis and septic shock. In the present study, we focused on the effects of TLR4 blockade on muscle wasting and muscle function in sepsis, which the clinical trials did not examine. Our finding that TAK-242 ameliorated muscle wasting, as measured histologically and functionally, suggests that TLR4 inhibition might improve skeletal muscle function in survivors, thereby improving their functional outcome and long-term survival. Our results thus provide a rationale to test the effects of TLR4 inhibitors on sepsis-induced muscle wasting and long-term survival in clinical trials.

There were several limitations in this study. First, as was the case in many previous studies^36,37,62^, our experiments examined the preventive effects of TLR4 inhibition by administering TAK-242 before LPS administration. Therefore, we cannot predict the effects of TLR4 inhibition on sepsis when the infection and systemic inflammatory response is ongoing. Nevertheless, our data have potential relevance for clinical practice, as previously noted^36^, because TLR4 inhibition may be a useful prophylactic treatment before major invasive surgical procedures that present a risk for infection and subsequent sepsis and skeletal muscle wasting^64,65^. According to reports by Sha *et al*.^61^ and Takashima *et al*.^32^, treatment with TAK-242 in combination with antibiotics 1 h after caecal ligation and puncture or *Escherichia coli* live bacterial challenge significantly suppressed serum levels of many inflammatory cytokines and increased the survival rates in mice. Second, this study used LPS only and did not investigate polymicrobial sepsis, such as that induced by caecal ligation and puncture. However, the LPS model used here is widely used to study muscle catabolism with a major inflammation component^19,20,30,31,59,60^. Third, because food and water were not withheld during the experiment, observed changes among mice treated with vehicle, LPS, or LPS and TAK-242 can be attributed to differences in food intake or nutritional status, as shown in Fig. 3D. Our experimental protocol included 2 days for monitoring muscle catabolism and weakness, and our Animal Care and Use Committees did not allow fasting for such a long period. We are also aware that other unmeasured factors, such as hypotension and hypothermia induced by LPS may have confounded our results. Nevertheless, we believe our data have potential relevance for clinical settings, because patients with endotoxemia and systemic inflammation are likely to become lethargic with a loss of appetite.

TLR4 can be activated by danger-associated molecular patterns^66^, such as HMGB1 and heat-shock proteins, which are known to induce muscle atrophy^67,68^. For example, a recent study^68^ showed that the systemic activation of TLR4 by cancer-released heat shock proteins 70 and 90 was responsible for the development and progression of muscle wasting in cancer. TLR4 also mediates insulin resistance^69,70^. which is associated with muscle protein degradation and cachexia^71,72^. Thus, TLR4-mediated muscle catabolism may take occur in many conditions associated with cachexia in addition to endotoxemia. Zhang *et al*.^73^ recently showed that the genetic inhibition of TLR4 successfully reversed Lewis lung carcinoma-induced muscle wasting in mice. Additionally, Schellekens *et al*.^74^ found that controlled mechanical ventilation caused a loss of myosin and increased cytokine expression in the diaphragm muscle of healthy C57BL/6 mice, but not of TLR4 knockout mice. Taken together, these observations suggest that our data might have significance in conditions beyond muscle wasting associated with endotoxemia. Further studies are warranted to test the effects of pharmacological TLR4 inhibition on skeletal muscle wasting in various pro-catabolic conditions.

## Conclusions

Using C2C12 myotubes *in vitro* and a mouse model of endotoxemia *in vivo*, we found that TAK-242, a selective inhibitor of TLR4-mediated signalling, blocked systemic and skeletal muscle inflammatory reactions, inhibited the activation of various proteolytic pathways, and ameliorated skeletal muscle wasting induced by LPS. These findings may prove helpful for the development of new therapeutic strategies to attenuate endotoxemia-induced muscle wasting.

## Methods

### Cell culture

Murine C2C12 myoblasts (RIKEN Cell Bank, Cell No. RCB0987, Tsukuba, Japan) were cultured in high-glucose Dulbecco’s modified Eagle’s medium (DMEM; Wako Pure Chemicals, Osaka, Japan) supplemented with 10% (vol/vol) foetal bovine serum (Equitech Bio, Kerrville, TX), 100 U/mL penicillin, and 100 μg/mL streptomycin (Wako) at 37 °C in a 5% CO_2_ atmosphere. When the cells reached 80% confluence, myoblast differentiation was induced by transfer to differentiation medium consisting of high-glucose DMEM, 2% heat-inactivated horse serum (Thermo Fisher Scientific, Waltham, MA), 100 U/mL penicillin, and 100 μg/mL streptomycin. LPS from *Escherichia coli* 026:B6 (Sigma Aldrich, St. Louis, MO) was dissolved in PBS and added to the differentiation medium at 0 and 24 h at a final concentration of 1 μg/ml. Unstimulated cells received the same volume of PBS. TAK-242 (Merck Millipore, Darmstadt, Germany) was dissolved in dimethyl sulfoxide (DMSO; Sigma Aldrich, final concentration: 0.1% vol/vol) and added to cells at a final concentration of 1 μM 1 h before the addition of LPS unless otherwise indicated. Control cells received an equal volume of DMSO at a final concentration of 0.1% vol/vol. TNF-α neutralizing antibody (goat polyclonal, Cat. No. AB-410-NA; R&D Systems, Minneapolis, MN) was added at 5 μg/mL in PBS at 1 h prior to the addition of LPS. These concentrations of TAK-242 and anti-TNF-α neutralizing antibody were used with reference to previous relevant studies^22,33,40,70^.

### Animal experiments

All experimental protocols were approved by the Institute of Animal Care and Use Committees at Fukushima Medical University (No. 30001) and Kobe university (No. P190801) and carried out according to relevant guidelines and regulations. Male C57BL/6 mice aged 8–12 weeks (Japan SLC, Hamamatsu, Japan) were quarantined in quiet humidified rooms on a 12 h light/dark cycle (7 am/7 pm) for at least 1 week before use. Mice were allowed access to standard rodent diet (CE7; Clea, Osaka, Japan) and water *ad libitum*. Food intake and body weight were measured every 24 h for 48 h. Muscle catabolism was induced by the i.p. injection of 1 mg/kg LPS, or an equal volume of vehicle (PBS) for controls, as previously described^19^. Mice were pretreated with TAK-242 (3 mg/kg) or vehicle (PBS containing 0.9% DMSO) by i.p. injection 1 h before LPS injection unless otherwise specified. These LPS and TAK-242 doses and administration times were selected based on several relevant articles^19,32,36,37,62^. Two days later, mice were euthanised and the TA muscles were collected, immediately frozen in liquid nitrogen, and stored at −80 °C until use.

### RNA isolation and real-time reverse-transcription polymerase chain reaction (qRT-PCR)

Total RNA was extracted from myotubes or muscle tissue using Isogen (Wako) according to the manufacturer’s protocol. First-strand cDNA synthesis and qRT-PCR was performed as described previously^22,75^. Sequences of the specific primers used were: atrogin-1/MAFbx, Fw: 5′-CACATTCTCTCCTGGAAGGGC-3′, Rv: 5′-TTGATAAAGTCTTGAGGGGAAAGTG-3′; MuRF1, Fw: 5′-CACGAAGACGAGAAGATCAACATC-3′, Rv: 5′-AGCCCCAAACACCTTGCA-3′; TNF-α, Fw: 5′-TACTGAACTTCGGGGTGATTGGTCC-3′, Rv: 5′-CAGCCTTGTCCCTTGAAGAGAACC-3′; IL-6, Fw: 5′-CCGGAGAGGAGACTTCACAG-3′, Rv: 5′-GGAAATTGGGGTAGGAAGGA-3′; and casein kinase 2a2 (CK2), Fw: 5′-GGAGGCCCTAGATCTTCTTG-3′, Rv: 5′-CGCGTTAAGACGTTTTGATT-3′. Detected levels of target mRNAs were calculated by the ΔΔCt method and normalised to CK2 in arbitrary units, using the StepOne Real-Time PCR System (Thermo Fisher Scientific).

### Western blot analysis

Muscle tissue and myotubes were homogenised and lysed, respectively, in RIPA buffer containing 50 mM Tris-HCl (pH 8.0), 150 mM NaCl, 1% Nonidet P-40, 0.5% sodium deoxycholate, and 0.1% sodium dodecyl sulphate supplemented with 1% protease inhibitor cocktail (Thermo Fisher Scientific) and incubated at 4 °C for 15 min. For phosphorylated proteins, 1% protease and phosphatase inhibitor cocktail (Thermo Fisher Scientific) were applied to RIPA buffer instead of protease inhibitor cocktail. The homogenates and lysates were then sonicated twice for 5 s each and centrifuged at 4 °C for 10 min at 15,000 × *g*. The supernatants were collected, and protein concentrations were determined using a protein assay kit (Bio-Rad, Hercules, CA) with bovine serum albumin (Sigma Aldrich) as a standard. Western blotting was performed as described previously with minor modifications^22^. In brief, equal amounts of protein (10–20 μg) were separated on 10% or 15% polyacrylamide gels and transferred to polyvinylidene difluoride membranes (Immobilon-P, Merck Millipore) using a wet transfer method. Membranes were blocked with 5% (wt/vol) non-fat dried milk and incubated for 1 h at room temperature with primary antibodies specific for MyHC (mouse monoclonal, Cat. No. 14-6503, Affymetrix, San Diego, CA; 1:500 dilution), LC3B (rabbit polyclonal, ab51520, Abcam, Cambridge, UK; 1:3000 dilution), MAFbx (mouse monoclonal, sc-166806; Santa Cruz Biotechnology, Santa Cruz, CA. 1:50 dilution) or β-tubulin (rabbit polyclonal, ab6046, Abcam; 1:1000 dilution). The other blots were incubated overnight at 4 °C with the following primary antibodies from Cell Signaling Technology (Beverly, MA): phospho (P)-p70 S6 kinase (Thr389) (rabbit monoclonal, #9234; 1:250 dilution), p70 S6 kinase (rabbit monoclonal, #2708; 1:500 dilution), P-Akt (Ser473) (rabbit monoclonal, #9271; 1:250 dilution) or Akt (rabbit monoclonal, #4685; 1:500 dilution). The blots were washed in PBS-Tween® 20 (0.05% vol/vol, Sigma-Aldrich) three times and probed with the corresponding horseradish peroxidase-conjugated secondary antibody (1:3000 dilution of either goat anti-rabbit IgG, sc-2004, Santa Cruz Biotechnology, or goat anti-mouse IgG, 62-6520, Thermo Fisher Scientific). Densitometric analysis of protein bands was performed using Clarity Western ECL Substrate (Bio-Rad) and ChemiDoc XRS Plus image analysis software (Bio-Rad). Protein expression is expressed as the densitometric ratio of protein normalised to β-tubulin.

### NF-κB assay

Nuclear extracts were prepared from C2C12 cells or TA muscles using a Nuclear Extract Kit (Active Motif, Carlsbad, CA). NF-κB (p65) DNA-binding activity was measured using a TransAM Enzyme-Linked Immunosorbent Assay (ELISA) kit (Active Motif) according to the manufacturer’s protocol. Sample absorbance at 450 nm was measured in a spectrophotometer (Multiskan GO, Thermo Fisher Scientific). NF-κB activity is expressed as the sample absorbance at 450 nm or fold increase over the vehicle-treated controls.

### Cell culture supernatant and plasma cytokine measurements

C2C12 myotubes were treated with DMSO (0.1% vol/vol) or TAK-242 (1 μM) and then with PBS or LPS (1 μg/mL) 1 h later. After 4 h cell culture, media were collected and centrifuged at 3000 rpm for 10 min at 4 °C. The supernatant was removed and stored at −80 °C until tested.

Mice were injected with vehicle or TAK-242 (3 mg/kg) and then with PBS or LPS (1 mg/kg) 1 h later. After 4 h, mice were anaesthetised by the i.p. injection of medetomidine (0.003%; Domitor, Nippon Zenyaku, Koriyama, Japan), midazolam (0.04%; Dormicum, Astellas Pharma, Tokyo, Japan), and butorphanol tartrate (0.05%; Vetorphale, Meiji Seika Pharma, Tokyo, Japan) dissolved in sterile 0.9% NaCl. Blood samples were withdrawn from the inferior vena cava using a 27 G needle (Terumo, Tokyo, Japan) into EDTA tubes, which were centrifuged at 3000 rpm for 15 min at 4 °C. The plasma layer was removed and stored at −80 °C until tested.

IL-6 and TNF-α concentrations in culture supernatant and plasma were determined using mouse IL-6 and TNF-α ELISA kits (Proteintech, Rosemont, IL), respectively, according to the manufacturer’s protocol. Absorbance at 450 nm was measured using a spectrophotometer (Thermo Fisher Scientific).

### Immunohistochemistry

C2C12 cells were seeded in poly-l-lysine-coated glass-bottomed dishes (Matsunami Glass, Osaka, Japan) and incubated in differentiation medium for 96 h. The cells were then transferred to differentiation medium alone or with LPS (1 μg/mL) or with LPS and TAK-242 (1 μM) for 48 h. At the end of the incubation, the cells were fixed with 4% (wt/vol) paraformaldehyde (Merck Millipore) and 0.2% (vol/vol) picric acid (Sigma Aldrich), washed in cold PBS, and incubated in blocking solution containing 2% (wt/vol) bovine serum albumin (Sigma Aldrich) and 5% (vol/vol) normal goat serum (Wako). The fixed cells were then incubated overnight at 4 °C in blocking solution containing anti-MyHC antibody (mouse monoclonal, Cat. No. 14-6503, Affymetrix; 1:500 dilution). After washing with PBS, the cells were incubated with Alexa Fluor 488-conjugated anti-mouse IgG (A-11029, Thermo Fisher Scientific; 1:400 dilution) for 40 min. Finally, after rinsing with PBS, the cells were covered with an antifade mounting medium containing 4′,6-diamidino-2-phenylindole (Vector Laboratories, Burlingame, CA). Fluorescence images were acquired using a confocal laser scanning microscope (Fluoview FV10i; Olympus, Tokyo, Japan).

Images were analysed for myotube width using the method of Menconi *et al*.^76^, with modifications. Briefly, the diameters of 97–114 myotubes from at least nine random fields were measured at three points along their length using ImageJ software version 1.39 (National Institutes of Health, Bethesda, MD), and averaged for each myotube. Data are expressed as the mean value and distribution of myotube widths.

The fusion index, defined as the number of nuclei in MyHC expressing myotubes divided by the total number of nuclei^77^, was also used as a morphological parameter of muscle differentiation. The number of nuclei in each myotube and the total number of nuclei were counted in five randomly selected fields using ImageJ software.

### Histology

Mice were injected i.p. with vehicle, LPS (1 mg/kg), or LPS and TAK-242 (3 mg/kg) as described above, and 2 days later, the mice were euthanised and perfused with 4% paraformaldehyde containing 0.2% picric acid using a peristaltic pump (Tokyo Rikakikai, Tokyo, Japan). The TA muscles were removed, embedded in paraffin, sectioned (5 μm thick), and stained with haematoxylin and eosin (H&E) solution (Wako) as previously described^78^. Sections were examined and images were captured using a light microscope (Olympus BX43) equipped with a camera (Olympus DP70). The cross-sectional area of myofibres was quantified with ImageJ software according to the method of Zhang *et al*.^79^. In brief, approximately 500 myofibres in each section were measured (five fields of view with approximately 100 myofibres per field). Data are expressed as the mean value and distribution of cross-sectional areas.

### Grip strength test

Forelimb grip strength was determined using a grip strength meter (MK-380Si; Muromachi Kikai, Tokyo, Japan) as previously described^80^ with minor modifications. In brief, mice were allowed to use their front paws to grab a horizontal bar mounted on the gauge, and the tail was slowly pulled back. The peak tension was recorded at the time the mouse released the grip on the bar. Measurements were repeated three times, and the mean of the three measurements was recorded. Grip strength was measured before and 48 h after the i.p. injection of vehicle, LPS (1 mg/kg), or LPS and TAK-242 (3 mg/kg).

### Statistical analysis

Data are presented as the means ± standard error (s.e.m.). Data were analysed by one-way analysis of variance (ANOVA) with post hoc analysis by Tukey’s honest significant difference test. Non-normal distributed data were analysed by the Kruskal-Wallis test. When a significant difference was detected by the Kruskal-Wallis test, Dunn’s post hoc tests were applied with Bonferroni correction. All analyses were performed using IBM SPSS Statistics for Windows, version 21.0 (IBM Corp., Armonk, NY). A p value < 0.05 was considered statistically significant.

## Supplementary information

Supporting Information.
