# Supplementary material for: TAK-242, a specific inhibitor of Toll-like receptor 4 signalling, prevents endotoxemia-induced skeletal muscle wasting in mice
Source: Sci Rep. 2020 Jan 20;10:694. doi: 10.1038/s41598-020-57714-3 (PMC6970997; doi:10.1038/s41598-020-57714-3)
Supplement: Supplementary file 1 — Supporting Information. [file 41598_2020_57714_MOESM1_ESM.pdf]

Supplementary Information

TAK-242, a specific inhibitor of Toll-like receptor 4 signalling, prevents endotoxemia-induced skeletal muscle wasting in mice

Yuko Ono[1,2], Yuko Maejima[2], Masafumi Saito[1], Kazuho Sakamoto[3], Shoichiro Horita[2], Kenju Shimomura[2], Shigeaki Inoue[1], Joji Kotani[1]

[1]Department of Disaster and Emergency Medicine, Graduate School of Medicine, Kobe University, Kobe, 650-0017, Japan
[2]Department of Bioregulation and Pharmacological Medicine, School of Medicine, Fukushima Medical University, Fukushima, 960-1295, Japan
[3]Department of Bio-Informational Pharmacology, School of Pharmaceutical Sciences, University of Shizuoka, Shizuoka, 422-8526, Japan

Supplementary Information

Supplementary Figure S1

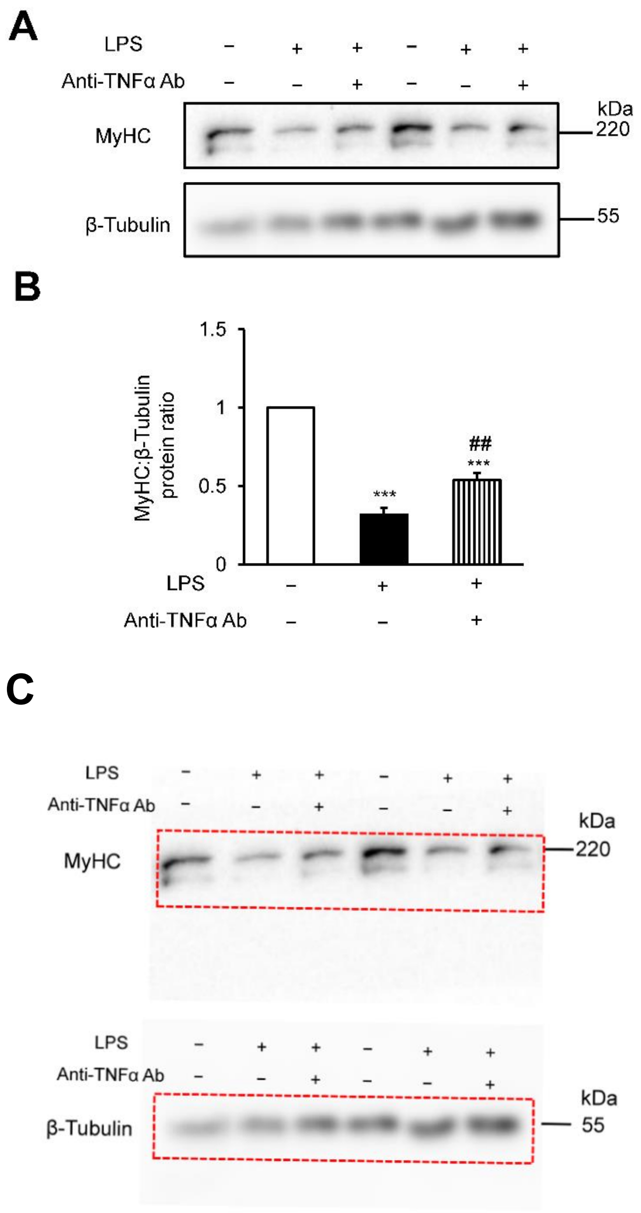

Figure S1. TNF-α-neutralizing antibody ameliorates LPS-induced MyHC protein loss in C2C12 myotubes.

(A, B) Western blot analysis (A) and quantification (B) of MyHC expression in C2C12 myotubes treated for 48 h with vehicle, LPS (1 μg/mL), or LPS and TNF-α neutralizing antibody (5 μg/mL). Data in (B) were normalised to β-tubulin protein levels, and the

ratio in vehicle-treated control cells was set to 1. N = 4/group. (C) Full length blots presented in Figure S1A. Dashed line represents the cropped region shown in Figure S1A. Data are presented as the mean ± s.e.m. ***$p < 0.001$ vs vehicle control; ##$p < 0.01$ vs LPS-treated group. P-values were derived from one-way ANOVA followed by Tukey's honest significant difference test.

Supplementary Figure S2

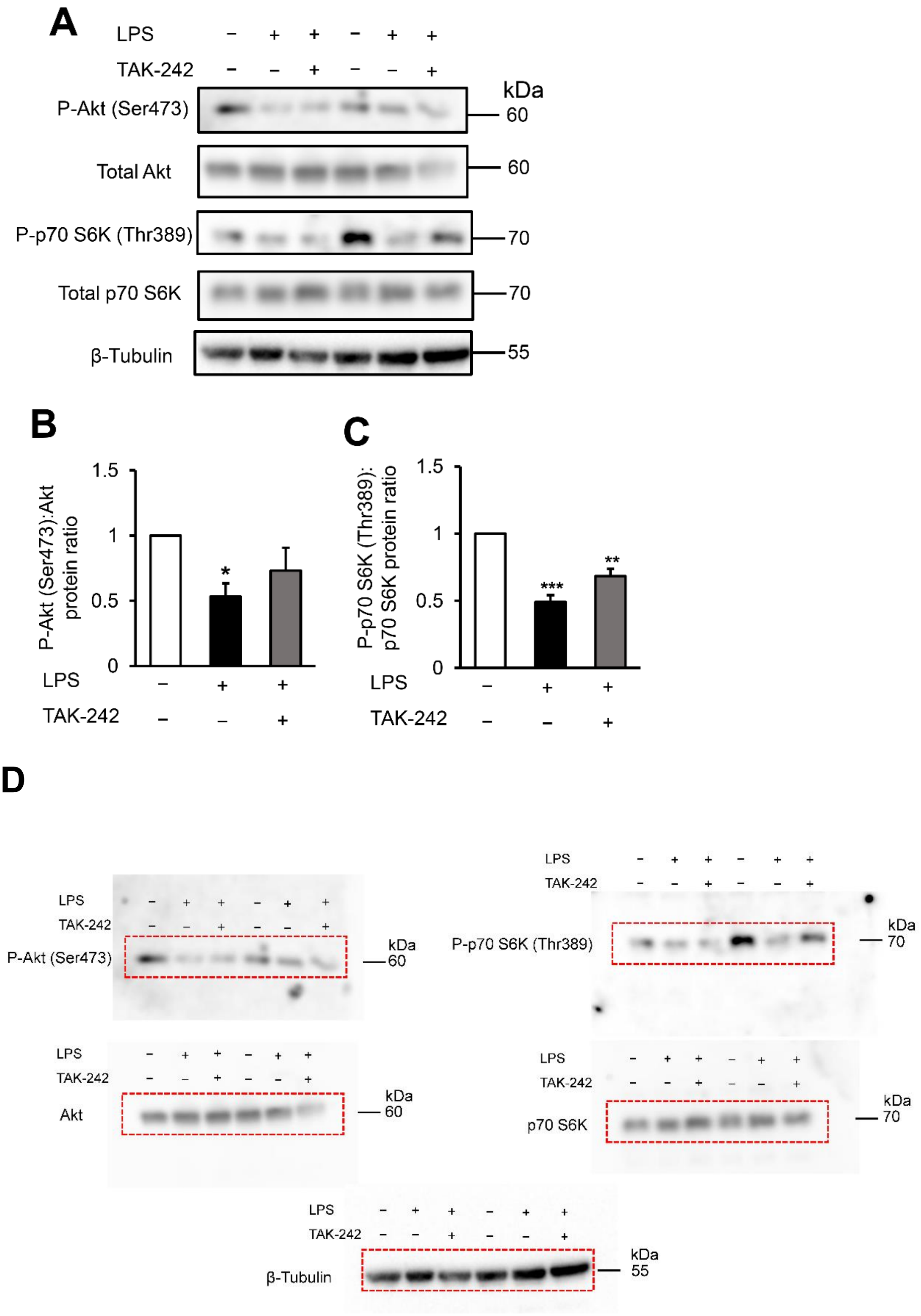

Figure S2. TAK-242 does not rescue decreased phosphorylation of Akt and p70 S6K in C2C12 myotubes induced by LPS.

(A-C) C2C12 myotubes were treated with vehicle (0.1% vol/vol DMSO) or TAK-242 (1 μM) and then with PBS or LPS (1 μg/mL) 1 h later. After 4 h, cells were lysed and subject to western blot analysis as described in the Methods. Western blot analysis (A) and quantification of Akt (B) and p70 S6K (C) phosphorylation in C2C12 myotubes. P-Akt (Ser473) and P-p70 S6K (Thr389) levels were normalised to total Akt and p70 S6K protein levels, respectively, and the ratio in vehicle-treated control cells was set at 1.0. N = 4–6/group. (D) Full length blots presented in Figure S2A. Dashed line represents the cropped region shown in Figure S2A. For all panels, data are presented as the mean ± s.e.m. ***p < 0.001, **p < 0.01, *p < 0.05 vs vehicle control. P-values were derived from one-way ANOVA followed by Tukey's honest significant difference test.

Supplementary Information

Supplementary Figure S3

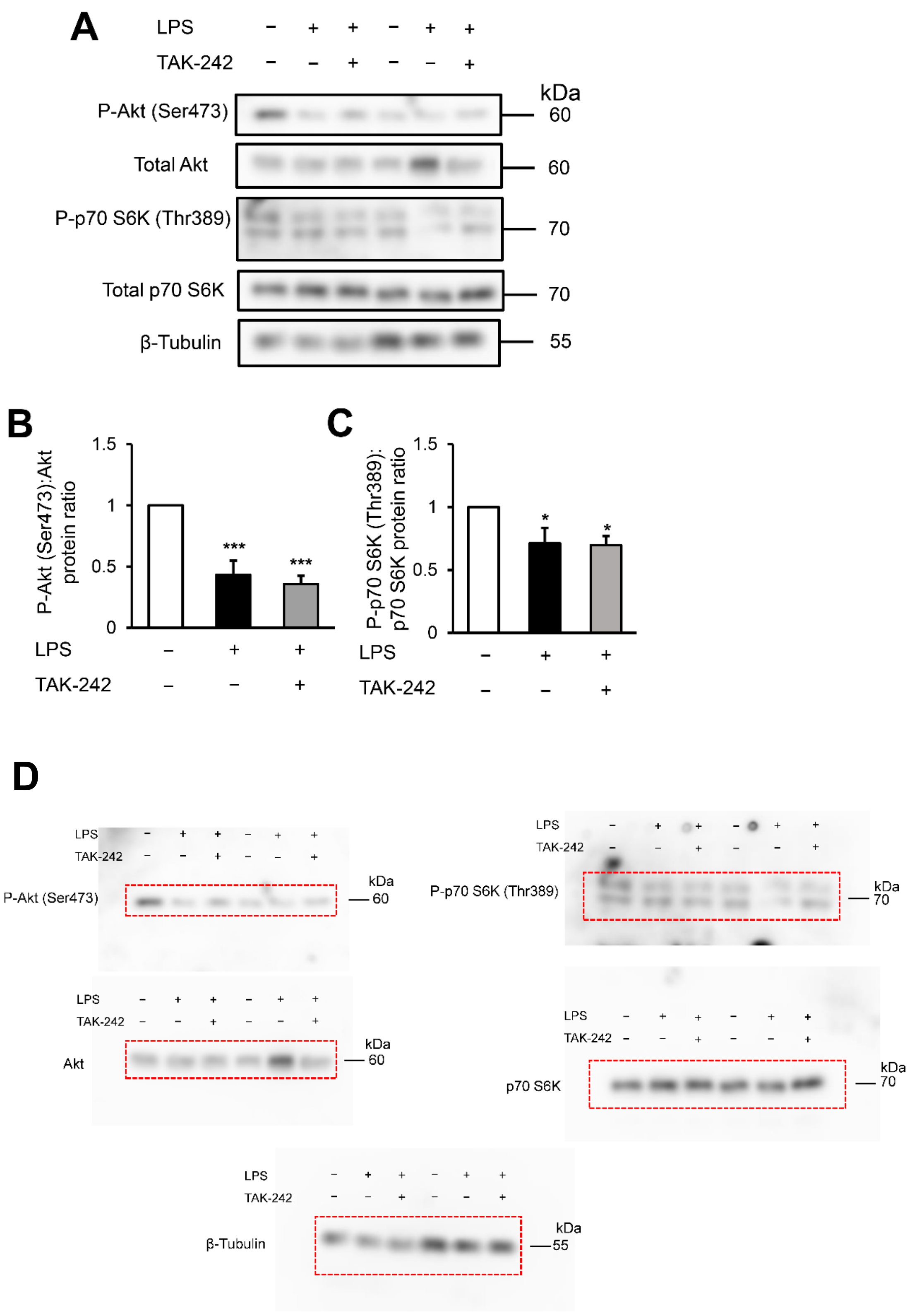

Figure S3. TAK-242 does not rescue decreased phosphorylation of Akt and p70 S6K in TA muscles of LPS-treated mice.

(A-C) Wild-type C57BL/6 mice (8–12-week-old males) were injected with vehicle (PBS containing 0.9% DMSO) or TAK-242 (3 mg/kg) and then with PBS or LPS (1 mg/kg) 1 h later. After 4 h, TA muscles were removed and lysed, then subject to western blot analysis as described in the Methods. Western blot analysis (A) and quantification of Akt (B) and p70 S6K (C) phosphorylation in TA muscles. P-Akt (Ser473) and P-p70 S6K (Thr389) levels were normalised to total Akt and p70 S6K protein levels, respectively, and the ratio in vehicle control-treated mice was set at 1.0. N = 5–8/group. (D) Full length blots presented in Figure S3A. Dashed line represents the cropped region shown in Figure S3A. For all panels, data are presented as the mean ± s.e.m. ***$p < 0.001$, *$p < 0.05$ vs vehicle control. P-values were derived from one-way ANOVA followed by Tukey's honest significant difference test.

Supplementary Information

Supplementary Figure S4

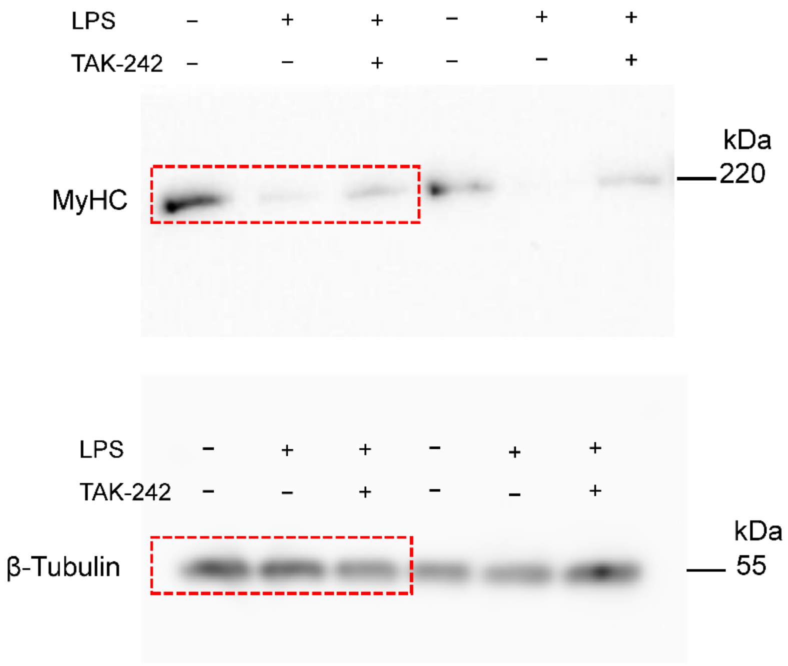

Supplementary Figure S4. Full length blots presented in Figure 1A. Dashed line represents the cropped region shown in Figure 1A.

Supplementary Information

Supplementary Figure S5

A

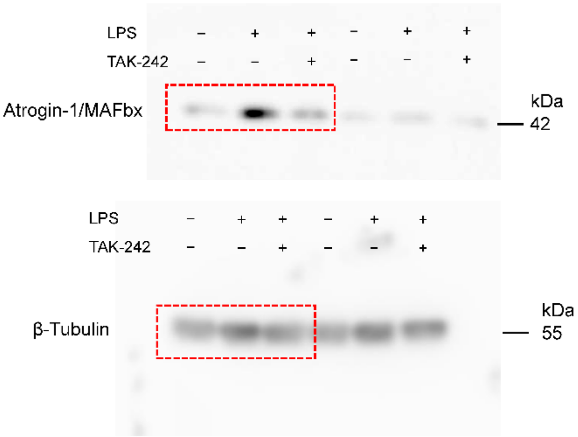

B

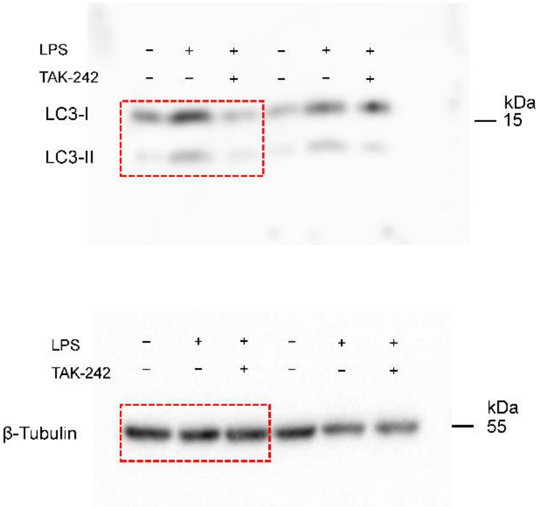

Supplementary Figure S5. Full length blots presented in Figure 2G (A) and 2K (B).

Dashed line represents the cropped region shown in Figure 2G and 2K.

Supplementary Information

Supplementary Figure S6

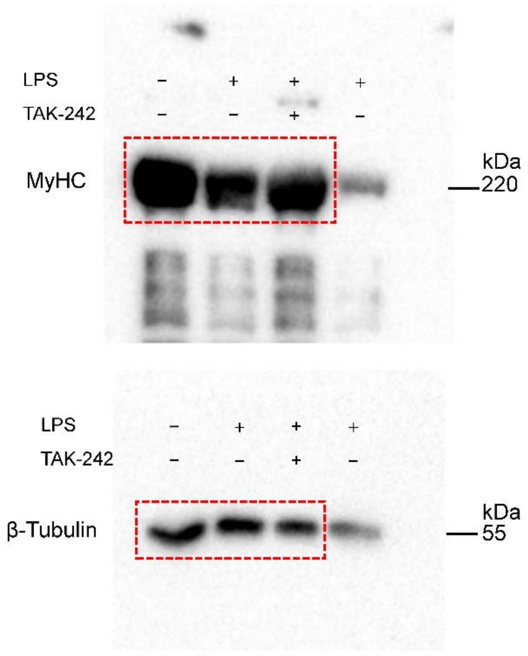

Supplementary Figure S6. Full length blots presented in Figure 3E.
Dashed line represents the cropped region shown in Figure 3E.

Supplementary Figure S7

A

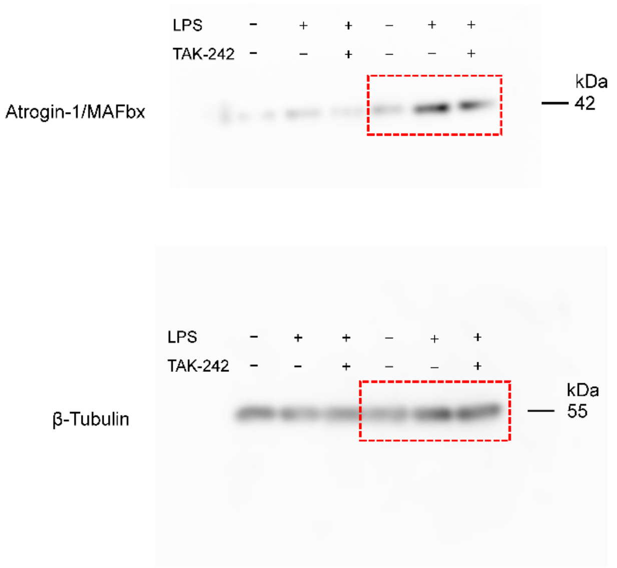

B

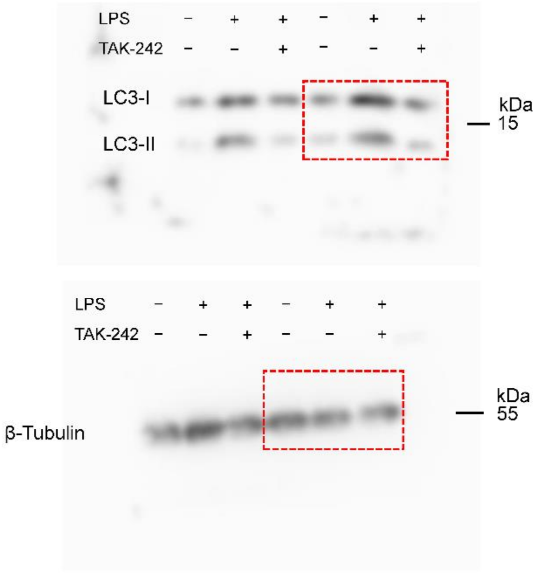

Supplementary Figure S7. Full length blots presented in Figure 4G (A) and 4K (B).

Dashed line represents the cropped region shown in Figure 4G and 4K.
